# Tissue repair capacity of bioceramic endodontic sealers in rat subcutaneous tissue

**DOI:** 10.1590/0103-6440202305161

**Published:** 2023-07-17

**Authors:** George Sampaio Bonates dos Santos, Ceci Nunes Carvalho, Rudys Rodolfo de Jesus Tavares, Paulo Goberlânio de Barros Silva, George Táccio de Miranda Candeiro, Etevaldo Matos Maia

**Affiliations:** 1 Post-Graduation Department, Universidade CEUMA, São Luis, Maranhão, Brazil.; 2 Christus University Center (Unichristus), Fortaleza, Ceará, Brazil

**Keywords:** Collagen Type I, Collagen Type III Silicate Cement, Subcutaneous Tissue, Mice

## Abstract

This study aimed to evaluate the tissue repair capacity of four bioceramic endodontic sealers by quantifying type I and III collagen fibers. The following sealers were tested: EndoSequence BC Sealer (Brasseler, Brasseler, Savannah, USA), Bio C Sealer (Angelus, Londrina, Brazil), Bioroot RCS (Septodont, Santa Catarina, Brazil), and Sealer Plus BC (MKLife, Porto Alegre, Brazil). Polyethylene tubes 1.5 mm in diameter and 1 cm in length containing the endodontic sealers were implanted in the subcutaneous tissue of five rats (*Rattus norvegicus albinus*, Wistar lineage). After 14 days, the animals were euthanized, and collagen fibers were quantified from the histological tissue sections. Given a non-normal distribution of the data, a gamma regression with log link function was employed and implemented through the generalized linear models module, was used to test whether there was a significant difference between the sealers. The pairwise comparison was performed using Least significant difference. There were significant differences between the sealers for type I (p=0.001), type III (p=0.023), and total collagen (p=0.002). Overall, Bioroot sealer was statistically superior to the other sealers, except in the analysis of type III collagen, in which there was no difference between the Bioroot sealer and Bio C Sealer sealer and the control group (p>0.05). Bioroot RCS bioceramic endodontic sealer stimulates a greater production of collagen.

## Resumo

Este estudo visou avaliar a capacidade de reparação de tecidos de quatro cimentos endodônticos biocerâmicos através da quantificação de fibras colágenas de tipo I e III. Foram testados os seguintes cimentos: EndoSequence BC Sealer (Brasseler, Brasseler, Savannah, EUA), Bio C Sealer (Angelus, Londrina, Brasil), Bioroot RCS (Septodont, Santa Catarina, Brasil), e Sealer Plus BC (MKLife, Porto Alegre, Brasil). Foram implantados tubos de polietileno de 1,5 mm de diâmetro e 1 cm de comprimento contendo os cimentos endodônticos no tecido subcutâneo de cinco ratos (*Rattus norvegicus albinus, linhagem Wistar*). Após 14 dias, os animais foram eutanasiados e as fibras colágenas foram quantificadas a partir de cortes histológicos do tecido. Diante de uma distribuição não-normal dos dados, uma regressão gama com função de ligação log, implementada por meio do módulo de modelos lineares generalizados, foi empregada para testar se havia diferença significativa entre os cimentos. A comparação dois a dois foi realizada utilizando Least significant difference. Houve diferença significativa entre os cimentos para os colágenos tipo I (p=0,001), tipo III (p=0,023) e colágeno total (p=0,002). No geral, o cimento Bioroot foi estatisticamente superior aos demais cimentos, com exceção na análise do colágeno tipo III na qual não houve diferença entre o cimento Bioroot e o cimento Bio C Sealer e o grupo controle (p>0,05). O cimento endodôntico biocerâmico Bioroot RCS foi capaz de estimular uma maior produção de colágeno.

## Introduction

One of the requirements for successful endodontic treatment is adequate sealing of the root canal using gutta-percha cones and endodontic sealer ^1^. A variety of endodontic filling sealers are available in the market, including those based on zinc oxide and eugenol, calcium hydroxide, glass ionomer, silicone, resin, and the most recently developed bioceramic sealers, resulting from the combination of calcium silicate and calcium phosphate ^2^.

Biocompatibility is the ability of a material or substance to elicit an appropriate host response in a specific application ^3^. Therefore, endodontic sealers should be biocompatible ^4^ because components present in the composition may induce irritation or persistent inflammation especially when extravasated in the periradicular tissues, which should be avoided ^5^. However, most sealers are toxic, especially when freshly prepared, and therefore should undergo tests to prove they may be safely used under clinical conditions ^6^. In addition to being biocompatible, endodontic sealers should be capable of helping to repair periapical tissue by inducing the recruitment of osteogenic and/or odontogenic cells surrounding the apical tissue.

Collagens form a family of around 30 proteins that are crucial structural molecules in the human body ^7^. The structure and remodeling of collagen in vivo are important for the healing of many human diseases, as well as for normal tissue development and regeneration. The specific properties of collagen matrices directly impact cell adhesion, propagation, and proliferation rates ^7^,

Collagen type I is expressed in the extracellular matrix, serve an important role in osteoblastic mineralization ^8^, and is characterized by the production of skin, bones, and tendons ^7^.

Type III fibers are precursors of the skin, muscles, and vessels, and are responsible for maintaining the structure of internal organs ^7^. Thus, the quantification of the density of types I and III collagen fibers combined with the sealer is a good indication for understanding if this could create a stimulating, compatible environment for the repair of periapical tissue.

Bioceramic-based materials have been tested for their properties and have shown good physicochemical properties, such as alkaline pH, biocompatibility, low cytotoxicity, good flow and radiopacity, antimicrobial activity, and adequate setting time ^9^ ^,^ ^10^. Another advantage is the release of calcium and phosphate ions, which induces bone tissue regeneration ^11^. However, few studies have evaluated the behavior of these sealers in relation to their stimulation of collagen fiber formation. Thus, the aim of the present study was to evaluate the tissue response in vivo in respect of four bioceramic sealers (EndoSequence BC Sealer, Bio C Sealer, Bioroot RCS, and Sealer Plus BC) with regard to the formation of types I and III collagen fibers in the subcutaneous tissue in rats. Accordingly, the null hypothesis tested in this study was that there would be no difference between the sealers with regard to the quantity of types I and III collagen fiber.

## Materials and methods

The present study was approved by the Ethics Committee on Animal Use of the School of Dentistry of Unicristus University, Fortaleza, CE, Brazil (protocol no. 008/20).

Five young adult male rats (*Rattus norvegicus albinus*, Wistar lineage) weighing 250-300g, aged approximately 75 days were used.

The sealers tested were the EndoSequence BC Sealer (Brasseler), Bio C Sealer (Angelus), Bioroot RCS (Septodont), and Sealer Plus BC (MKLife). The manufacturers, compositions, and proportions of the materials used in this study are listed in Box 1.

**Box 1:**
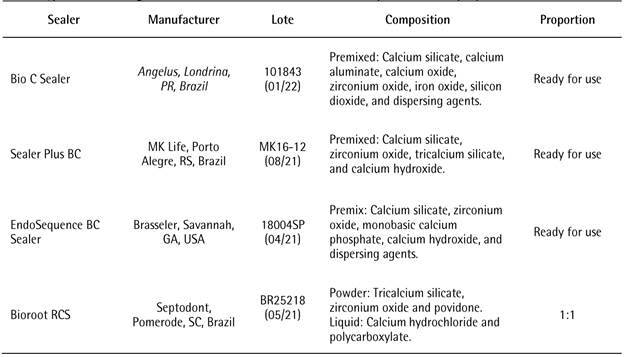
Type of obturating sealer used, manufacturers, chemical compositions, and proportions.

The sealers were prepared according to the manufacturer’s instructions. While the sealers EndoSequence BC Sealer (Brasseler), Bio C Sealer (Angelus), and Sealer Plus BC (MKLife) are ready for use, the Bioroot RCS (Septodont) required manipulation. For this, a portion of the powder, collected with the spoon supplied in the box, was placed on a glass plate (50x50x4mm). Five drops of liquid were poured over the powder. Using a spatula 24 (Golgran Ind. Com. Instr. Odontológicos, São Caetano do Sul, SP, Brazil) the powder was progressively mixed with the liquid until obtaining a smooth paste.

The sealers were inserted, soon after manipulation, in polyethylene tubes of approximately 1 cm in length and 1.5 mm in diameter, obstructed at one of the extremities using a hot needle holder, then implanted in the dorsal subcutaneous tissue of the animals. Five tubes were implanted per animal (four containing the sealers and one without material).

For tube implantation, animals were anesthetized with an intraperitoneal injection of a mixture of 80 mg/kg 10% ketamine hydrochloride (Alfasan, Woerden, Netherlands) and 20 mg/kg 2% xylazine hydrochloride (Alfasan, Woerden, Netherlands). Dorsal trichotomy was performed manually in five areas of approximately 10 cm^2^. Disinfection was performed using a 2% chlorhexidine solution. Five 2 cm long incisions were made on the backs of the animals. Using blunt-tip scissors, lateral openings were made in the subcutaneous tissue, providing five surgical cavities shown in quadrants equidistant from the center of the animals' backs. Tubes filled with the materials were immediately inserted into the surgical cavities parallel to the incision.

The incisions were closed with 3-0 silk thread (Supa, Tehran, Iran), and the region was again disinfected with a 2% chlorhexidine spray. All animals were euthanized after 14 days by an overdose of xylazine and ketamine (160 and 80 mg/kg). The areas of the tubes, along with 1 cm of tissue around the implant, were excised and fixed in 10% buffered formalin (Merck, Darmstadt, Germany) for 24 h.

The polyethylene tubes were then removed from the samples, and the remaining surrounding tissue was packed in paraffin blocks and processed for histological analysis. Three sections were obtained per sample, each measuring 3 µm in thickness, were placed on glass slides and deparaffinized in an oven at 60°C for 3 h in three xylene baths (5 min). After rehydration in a decreasing alcohol series, the slides were incubated in picrosirius solution (Williams & Wilkins, Baltimore, USA) for 30 min, washed quickly in two baths of 5% hydrochloric acid, counterstained with Harris hematoxylin for 45 s, and mounted with Entellan®. Five fields (200x) were selected and photographed in a conventional way and under polarized light using a camera (U-TV0.63 XC, Olympus ®) coupled to a BX43 microscope (Olympus ® with Olympus Soft Imagining LCMicro software) at 400x magnification, and exported to ImageJ® (National Institute of Health, Maryland, USA).

### Quantitative collagen fiber analysis

**Figure 1:**
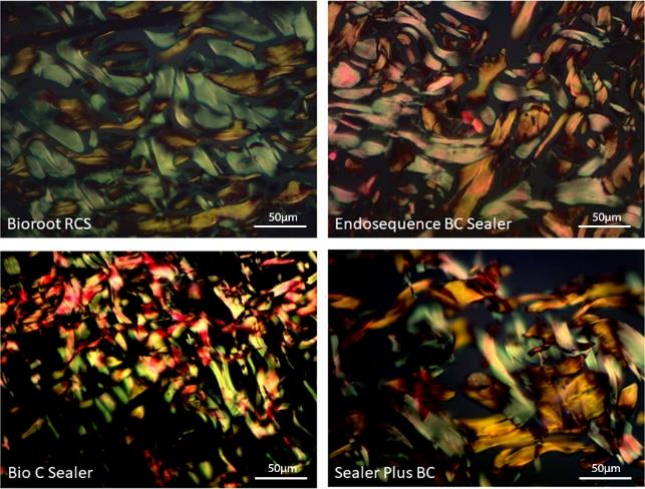
Representative photomicrographs of a 3 µm histological section at 400x magnification of a fibrous capsule stained in Pricosirius Red.

The areas of connective tissue of the subcutaneous tissue of the rats were subjected to picrosirius red analysis to verify the quantity and typification of collagen deposition. This technique confers a reddish coloration to collagenized areas, and light polarization suggests a possible distinction between collagen types through yellowish-red and whitish-green birefringence. For the analysis of total collagen, the photomicrographs were evaluated using ImageJ® software (http://rsbweb.nih.gov/ij/) after calibration of the images using the Color Threshold command (Image > Adjust > Color Threshold) in the RGB function for the colors red (minimum 71 and maximum 255), green (minimum 0 and maximum 69), and blue (minimum 0 and maximum 92) (Figure 1).

Bioroot RCS - Predominantly thick collagen fibers with, for the most part, a reddish-yellow birefringence, arranged horizontally, and fibers with a whitish-green birefringence can be seen interspersed with them.

EndoSequence BC Sealer - Collagen fibers are sometimes arranged lengthwise and sometimes crosswise, with a subtle predominance of fibers with reddish-yellow birefringence. Predominantly thick, interspersed fibers. BioCSealer - Thinner collagen fibers, though predominantly composed of fibers with reddish-yellow birefringence. The fibers are arranged in an interspersed fashion and are contiguous with one another.

Sealer Plus BC - Thick fibers, though mainly with whitish-green birefringence, aligned horizontally, and fibers with a reddish-yellow birefringence can be seen interspersed with them.

For polarized images, the same protocol was performed by adjusting the colors in the RGB function to red (minimum 0 and maximum 255), green (minimum 0 and maximum 255), and blue (minimum 0 and maximum 32). After adjustment, the images were converted to an 8-bit color scale (Image > Type > 8-bit) and binarized (Process > Binary > Make Binary). The percentage of collagen area marked reddish-yellow relative to the area marked in red was then measured. The green-white area was obtained using a process similar to that described above, by changing the RGB color channels to red (minimum 0 and maximum 65), green (minimum 0 and maximum 255), and blue (minimum 0 and maximum 255) ^12^.

All the analyses were conducted by a blinded, previously calibrated pathologist (kappa = 0.872).

Thicker, strongly birefringent collagen fibers were stained in shades of yellow to red, suggesting type I collagen, whereas thinner, more dispersed, weakly birefringent fibers were stained green, suggesting type III collagen.

### Statistical Analysis

To evaluate whether there was a statistically significant difference between the different types of endodontic sealers in relation to the percentage of collagen types I and III and total collagen and in the face of a non-normal distribution of data (Shapiro-Wilk test, p<0.05), a gamma regression with the log-link function was employed and implemented in the generalized linear model’s module (GZLM) of the software IBM SPSS Statistics for Windows v.26 (IBM Corp., Armonk, N.Y., USA) ^13^. Gamma regression, since it has a lower Akaike's information criterion (AIC) and Bayesian information criterion (BIC), was chosen after analyzing the histogram of the data frequency distribution. A two-by-two comparison was performed using the least significant difference. The significance level used was 5%.

## Results

Table 1 shows the mean percentage values and the standard deviation of the amount of collagen type I, type III, and total collagen according to the sealer evaluated and the control group.

Figure 1 shows examples of histological sections for each type of sealer tested, exhibiting type I and III collagen fibers.

**Figure 2:**
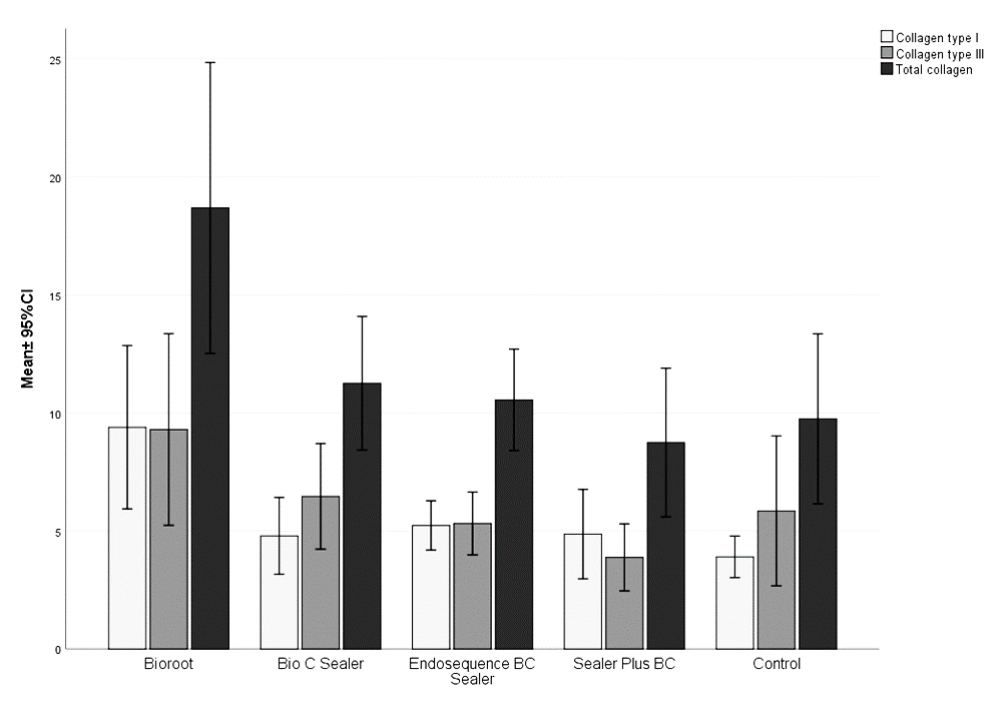
Mean percentage values (± 95% confidence interval) for collagen type I, III, and total collagen according to the type of sealer evaluated.

Figure 2 shows the column graphs with the respective 95% confidence intervals for the mean percentage of collagen type I, type III, and total collagen according to the sealer evaluated and the control group.

The mean collagen values were higher for the Bioroot sealer, regardless of the type of collagen analyzed.

There were significant differences between the sealers for type I (p=0.001), type III (p=0.023), and total collagen (p=0.002). Table 1 shows a two-by-two comparison by means of horizontal superscript letters. Different letters represent statistically significant differences (p<0.05).

In general, Bioroot sealer was statistically superior to the other sealers, except in the analysis of collagen type III, in which there was no difference between the Bioroot sealer, Bio C Sealer sealer, and the control group (p>0.05).

**Table 1:** Mean (±standard deviation) and median percentage values of collagen type I, III, and total collagen.

	Bioroot RCS	Bio C Sealer	EndoSequence BC Sealer	Sealer Plus BC	Control	p-value
Type I Collagen	9.4% (±7.80%) ^A^	4.79 (±3.85) ^B^	5.23 (±2.41) ^B^	4.87 (±3.68) ^B^	3.90 (±1.82) ^B^	0.001*
Type III Collagen	9.30 (±9.16) ^A^	6.47 (±5.30) ^AB^	5.32 (±3.08) ^B^	3.88 (±2.76) ^B^	5.85 (±6.59) ^AB^	0.023*
Collagen Total	18.69% (±13.91%) ^A^	11.26 (±6.70) ^B^	10.56 (±4.97) ^B^	8.75 (±6.12) ^B^	9.75 (±7.48) ^B^	0.002*

* p<0.05 = significant difference. Different horizontal letters indicate statistically significant differences.

## Discussion

The null hypothesis was rejected. Among the bioceramic sealers, there was a significant difference in the quantity of collagen fiber, both for type I and type III collagens and total collagen.

Implantation in rat subcutaneous tissue is one of the most widely used tests for determining the type and development of local reactions induced by endodontic sealers. Generally, rats are used because they are less susceptible to infection after surgery, are economically viable, and present a plausible model for determining the histocompatibility of materials ^14^.

Around the polyethylene tubes filled with endodontic sealers implanted in the dorsum of rats, a fibrous capsule and granulation tissue may form, which may indicate tissue tolerance ^15^. Light microscopy was used to observe collagen in this material because it provides a morphological evaluation of the characteristics of collagen as a starting point for the evaluation of biological responses to endodontic sealers ^7^. The collagen fiber formation process indicates the healing process's evolution ^15^.

The results of this study demonstrated the existence of differences in tissue repair among bioceramic sealers. The Bioroot RCS sealer (Septodont) was the only one that showed significantly higher amounts of type I collagen and total collagen than the other groups and was precisely the strongest collagen, linked to the final phase of tissue healing ^7^. The expression of type 1 collagen creates a microenvironment that favors the recruitment and differentiation of osteo-odontogenic stem cells by inductive signals. Since this marker is an indicator of stem cell homeostasis, also represents a useful readout to evaluate in vitro the biocompatibility of root canal sealers ^16^.

The difference between the BioRoot RCS (Septodont) and other sealers may be associated with the composition of the sealer, which plays an important role in biocompatibility ^17^. BioRoot RCS (Septodont) is a powder/liquid hydraulic tricalcium silicate-based sealer. The powder contains tricalcium silicate, povidone, and zirconium oxide, the liquid is an aqueous solution of calcium chloride and polycarboxylate. BioRoot RCS has been reported to induce in vitro the production of angiogenic and osteogenic growth factors by human periodontal ligament cells ^18^, moreover, it has lower cytotoxicity than other conventional root canal sealers, and may induce hard tissue deposition ^19^.

BioRoot RCS has been shown to have the ability to nucleate carbonated apatite deposits in relation to its prolonged ability to release calcium ions and to basify the environment ^20^. The prolonged release of calcium ions has been demonstrated to be a key factor to promote endodontic and periodontal tissue regeneration ^20^, biocompatibility, and bioactivity ^21^. Furthermore, Jeanneau et al. ^22^ demonstrated this sealer's anti-inflammatory effects and tissue regeneration potential with the stimulation of fibroblasts and beta 1 growth factors. These factors may also be related to its ability to stimulate higher collagen production, as observed in the present study.

In addition, Bioroot RCS (Septodont) has been shown to influence cell metabolism, with slight cytotoxicity and excellent biocompatibility at all concentrations, either as a freshly prepared material or with a stabilized setting time. Direct contact with cells did not affect cell vitality, morphology, and growth ^17^ ^,^ ^23^. Furthermore, Dimitrova-Nakov et al. ^16^ showed that mouse dental pulp exposed to Bioroot RCS sealer continuously displayed the same morphology as control cells, and the cell sheet remained uniform.

In this study, there was no significant difference in total collagen between the Bio C Sealer (Angelus), EndoSequence BC Sealer (Brasseler), Sealer Plus BC (MKLife), and the control group. A similar result was observed by Hoshino et al. ^24^, who, despite verifying the occurrence of a gradual increase in total collagen (7, 15, 30, and 60 days) in relation to bioceramic sealers, did not find a significantly different collagen amount from the control group.

It has been shown that the Bio C Sealer (Angelus) presents good cytocompatibility in terms of viability, migration, morphology, cell attachment, and mineralization capacity ^25^, and is biocompatible and safe for use in close contact with periapical tissue ^12^. EndoSequence BC Sealer sealer (Brasseler) has shown better cytocompatibility than MTA Fillapex (Angelus, Londrina, Brazil) ^26^, while Sealer Plus BC sealer (MKLife) was less cytotoxic to L929 (fibroblastic cells) when a less dilute concentration was used, and was more biocompatible than MTA Fillapex (Angelus) and AH Plus (Dentsply) ^27^. Apparently, the beneficial properties of these sealers were not sufficient to increase collagen production.

It is important to establish the setting conditions for the biological properties of the sealer. There are differences in cytotoxicity and biocompatibility between fresh and hardened sealers ^28^ ^,^ ^29^.

The release of unconverted monomers may play a role in the cytotoxicity of sealers that have not yet been established, whereas in conditions where sealer setting has already occurred, a residual toxic effect can be expected. However, this condition seems to be more plausible for resin sealers than for ready-to-use bioceramic sealers ^30^. In the present study, the material was inserted while still being fresh. From a clinical point of view, the use of freshly mixed sealers is relevant because these materials are applied when introduced into the root canals, allowing them to come into contact with the periapical tissues ^18^.

Regarding biocompatibility, the Bioroot RCS sealer (Septodont) showed better results than other epoxy resin-based or methacrylate-based sealers ^31^ or zinc oxide-eugenol-based sealers ^18^, and was also better than other calcium silicate-based sealers ^17^.

Under the conditions of this study, the Bioroot RCS bioceramic endodontic sealer stimulated increased collagen production.
